# De Novo Mutations Resolve Disease Transmission Pathways in Clonal Malaria

**DOI:** 10.1093/molbev/msy059

**Published:** 2018-05-01

**Authors:** Seth N Redmond, Bronwyn M MacInnis, Selina Bopp, Amy K Bei, Daouda Ndiaye, Daniel L Hartl, Dyann F Wirth, Sarah K Volkman, Daniel E Neafsey

**Affiliations:** 1 Broad Institute of MIT and Harvard, Cambridge, MA; 2 Harvard T.H. Chan School of Public Health, Boston, MA; 3 Department of Parasitology, Faculty of Medicine and Pharmacy, Cheikh Anta Diop University, Dakar, Senegal; 4 Department of Organismic and Evolutionary Biology, Harvard University, Cambridge, MA; 5 Department of Nursing, School of Nursing and Health Sciences, Simmons College, Boston, MA, 02115

**Keywords:** genomic epidemiology, de novo mutation, malaria, transmission networks

## Abstract

Detecting de novo mutations in viral and bacterial pathogens enables researchers to reconstruct detailed networks of disease transmission and is a key technique in genomic epidemiology. However, these techniques have not yet been applied to the malaria parasite, *Plasmodium falciparum*, in which a larger genome, slower generation times, and a complex life cycle make them difficult to implement. Here, we demonstrate the viability of de novo mutation studies in *P. falciparum* for the first time. Using a combination of sequencing, library preparation, and genotyping methods that have been optimized for accuracy in low-complexity genomic regions, we have detected de novo mutations that distinguish nominally identical parasites from clonal lineages. Despite its slower evolutionary rate compared with bacterial or viral species, de novo mutation can be detected in *P. falciparum* across timescales of just 1–2 years and evolutionary rates in low-complexity regions of the genome can be up to twice that detected in the rest of the genome. The increased mutation rate allows the identification of separate clade expansions that cannot be found using previous genomic epidemiology approaches and could be a crucial tool for mapping residual transmission patterns in disease elimination campaigns and reintroduction scenarios.

## Introduction

The reconstruction of transmission networks by sequencing pathogen samples is a powerful technique that has become increasingly routine in the field of genomic epidemiology. By identifying infections with shared de novo variation or the shortest genetic distances between samples, epidemiologists are able to reconstruct detailed transmission networks in viral and bacterial pathogens such as flu (Neher and Bedford 2015), MRSA (Harris et al. 2010; Popovich et al. 2016) or Ebola (Gire et al. 2014; Park et al. 2015). Assaying de novo mutations has proven to be a useful strategy in settings where epidemiological data is limited or in situations where the actual transmission pathways are difficult to identify, and it is applicable to any *measurably evolving population* (as defined by Drummond et al. 2003). Using de novo genetic variation to track pathogen population movement has enabled the identification of disease reservoirs underlying reemergence (Eyre et al. 2013); assessed the relative contribution of local versus imported infections in a given setting (Azarian et al. 2016); and allowed the identification of the rate and times of transmission across national borders (Faria et al. 2017; Metsky et al. 2017).

Despite the relevance of all these scenarios to malaria transmission and the potential impact on elimination efforts, inference of transmission networks based on de novo variation has not been attempted. Genomics-based transmission analyses in malaria has have thus far relied on standing variation (Nkhoma et al. 2013; Daniels et al. 2015). However, these approaches require the reassortment of common alleles via sexual outcrossing and will not show discriminatory power in regions where transmission rates are too low (in which the parasite predominantly mates with itself) or in situations where malaria has been reintroduced to a region (resulting in an entirely clonal outbreak). Indeed, even within regions with ongoing outcrossing, clonal outbreaks persisting across multiple years have been found (Daniels et al. 2015); as disease-endemic countries approach elimination these situations are likely to become more common.

Several challenges prevent the direct application of transmission network reconstruction methodologies in *Plasmodium*. In addition to complications introduced by sexual outcrossing, the larger genome of *P. falciparum* and a slow mutation rate relative to prokaryotic or viral pathogens reduces our ability to accurately resolve de novo mutations between individuals. The most rapidly mutating loci are found within low complexity sequence, and commonly within short tandem repeats (STRs). Mutation rates for STRs can be as high as 3.77 × 10^−2^ mutations/locus/generation (Chenet et al. 2015), many orders of magnitude higher than the estimated rate for SNPs in non-STR sequence (∼1.07 × 10^−9^ substitutions/locus/generation [Lemieux 2015]). STR mutations are themselves associated with increased mutation rates in other organisms (Lenz et al. 2014) and low-complexity sequences in *P. falciparum* show greatly increased evolutionary rates compared with high-complexity flanking sequences (Zilversmit et al. 2010).

These highly mutable, low complexity regions within the *P. falciparum* genome are difficult to access using conventional short-read sequencing approaches. Commonly used sequencing formats (100 bp paired-end reads within ∼300 bp fragments) prohibit high quality alignments in repetitive sequence, particularly when the size of the repeat region approaches or exceeds the read length. Where insertion/deletion (INDEL) mutations are found within STRs they can be impossible to detect, as insufficient nonrepetitive flanking sequence around the variant can lead to incorrect partial alignments or a complete failure to map reads (Narzisi and Schatz 2015).

By spanning long repeats, longer read lengths will greatly improve our ability to analyze variation in low complexity genomic regions (including SNPs, STR modifications and non-STR associated INDELs) and may, along with associated algorithmic advances in variant calling, enable studies of de novo variation in eukaryotes (Weisenfeld et al. 2014). Applying such an approach to clinical samples representing clonal expansions in Senegal, we show that de novo mutation can be reliably called in a eukaryotic parasite at a specificity that makes it amenable for determining phylogenetic relationships. We further demonstrate that *P. falciparum* can be considered a *measurably evolving population* (i.e., a population that can be sampled at different points in time generating a statistically significant number of genetic differences—Drummond et al. 2003) and is therefore a suitable subject for transmission network reconstruction. The wider use of these techniques would greatly increase the resolution of genomic epidemiology studies in malaria and could have a dramatic impact on efforts to eliminate the disease.

## Results

To increase our ability to detect rapidly arising mutations in the *P. falciparum* genome, a modified library preparation method was employed in which large volumes of DNA were prepared without a PCR step (minimizing the introduction of amplification errors); 250 bp Illumina reads were generated on a size-selected 450 bp fragment (ensuring overlapping reads that could be joined into one long fragment); these reads were genotyped with DISCOVAR, an algorithm designed specifically for this data type (Weisenfeld et al. 2014), as well as GATK HaplotypeCaller (DePristo et al. 2011). The extent and accuracy of these techniques were assessed using laboratory isolates of well-defined parasite clones, each with an associated genome assembly. These techniques were then applied to patient samples collected from a single clinic in Thiès, Senegal representing three separate clonal expansions circulating in the region. These clonal samples were genotyped using a 24 SNP barcode (Daniels et al. 2015) and were previously determined to be identical by fixed locus genotyping of commonly genotyped polymorphic genes (*MSP1/MSP2/TRAP/CSP*; A. Bei, *personal communication*).

### 250-bp Illumina Reads Increase Genotype Specificity in Low-Complexity Regions

#### Genome Accessibility

**Fig. 1.**
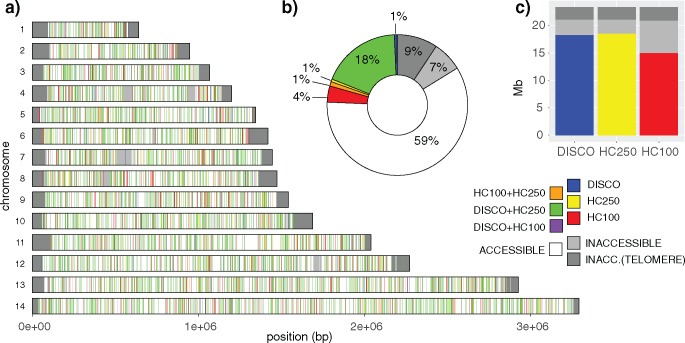
Genome accessibility was assessed based on in silico comparisons of two *P. falciparum* genome assemblies. Variants were called from reads simulated from the Pf_Dd2 reference and aligned to the Pf3D7_v3 reference and assessed as correct or incorrect based on a 200 bp flanking region on either side. Blocks of 1 kb across the genome were defined as accessible only if they had a read coverage within two standard deviations of the median and if they had called all variants accurately. Comparisons were made between DISCOVAR and HaplotypeCaller with both 250 bp (HC250) and 100 bp reads (HC100). Subplots show (*a*) the genome-wide distribution of accessible blocks, (*b*) the proportions accessible to combinations of callers, and (*c*) the overall genome area accessible to each caller. The strong overlap of HC250 and DISCOVAR calls suggests that the majority of this improvement derives from the use of longer reads; accessibility increased from 64.0% with HC100 to 78.4% with DISCOVAR and 79.3% with HC250. 10.8 Mb of low complexity sequence was identified by DustMasker, of which 80.8% 81.4%, and 65.4% was found to be accessible by DISCOVAR, HC250, and HC100, respectively.

The effect of read length and calling algorithm on genome accessibility was assessed by comparison of DISCOVAR (which is restricted to 250 bp reads) and GATK HaplotypeCaller with both 100 bp (hereafter HC100) and 250 bp reads (HC250). To assess genome accessibility we generated an in silico library from one genome assembly (Dd2) aligned this to another reference genome assembly (3D7) and assessed our ability to accurately reconstruct the second reference genome. A greater proportion of the *P. falciparum* genome was found to be “accessible” to DISCOVAR (78.4%) and HC250 (79.3%), than was accessible to HC100 (64%), indicating the importance of longer reads. This difference in accessibility represents >3.5 Mb: the majority of the genome outside of the mitotically recombining telomere and subtelomere sequence and intercalary heterochromatin (Flueck et al. 2009; see fig. 1). As expected, low-complexity regions also show a marked improvement in accessibility; of 10.8 Mb of sequence identified as low-complexity by DustMasker (Morgulis et al. 2006), 80% is accessible using either DISCOVAR or HC250 compared with 65% accessible using HC100.

#### Marker Validation

Following the in silico analysis of genome accessibility we assessed specificity and sensitivity of all three genotyping approaches by comparing a sequenced DD2-2D4 sample to a validated set of genotype calls from the Pf-Crosses variant set (Miles et al. 2016; Pf-Crosses data available at https://www.malariagen.net/data/pf-crosses-1.0). Receiver-Operator Characteristic (ROC) curves were generated for variants in regions that were accessible to all callers (the “core” genome) and variants found in the additional genome accessible to that caller (the “extended” genome; supplementary fig. S1, Supplementary Material online]. Specificity rates were similar across all callers, with DISCOVAR being the most specific, albeit at a significant cost to sensitivity (DISCOVAR precision 0.34/sensitivity 0.39; HC250 0.28/0.87; HC100 0.31/0.88); these results were similar when we included the extended genome (DISCOVAR 0.33/0.38; HC250 0.31/0.87; HC100 0.28/0.86) suggesting that there is little drop in specificity in low-complexity regions of the genome. It is notable that the ROC curves for HC250 and DISCOVAR show “false positive” variants even at the highest VQSLOD scores and in all regions of the genome, which likely represent real variants that were not callable with the 100 bp reads that were used for the Pf-crosses study.

### Higher Specificity Genotyping Produces Robust Phylogenetic Inferences

**Fig. 2.**
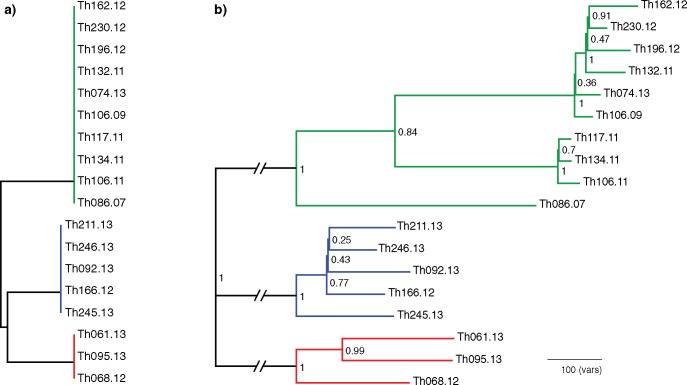
Phylogenetic resolution of parasite samples using standing variation versus de novo variants. (*a*) Maximum parsimony tree of standing variation (24-SNP molecular barcode—Daniels et al. 2015). The last two numerals in sample names indicate the year of collection. Clades are resolved, but samples within clades are indistinguishable. (*b*) More than 3,000 de novo variants were called via DISCOVAR that segregated the individuals into three IBD clades. Samples within clades 24 (red), 26 (blue), and 29 (green) were separated by 622, 828, and 1858, variants, respectively, with the closest individuals (Th106.09/Th074.13) distinguished by 88 de novo variants. Phylogenetic trees were calculated using 15276 SNPs and 11420 INDELs using maximum parsimony. Numbers on nodes indicate bootstrap support. Our ability to discern phylogeny using only de novo variants was high with bootstrap values above 0.5 for all nodes subtending samples collected at >1 year apart.

We prioritized specificity for subsequent analyses given the motivating application for these data was distance-based transmission network reconstruction methods, which are sensitive to false positive calls. Using the combination of 250 bp reads and DISCOVAR variant calls, we genotyped samples from a clinical isolate collection from Senegal, representing three clades (24, 26, and 29—Daniels et al. 2015). Samples within these clades were indistinguishable by previous techniques and represented independent infections collected over multiple years. To maximize specificity, DISCOVAR calls were produced independently from each of two lanes of sequencing data, allowing discordant genotypes to be discarded and further reducing the absolute abundance of false positive calls. A phylogeny was calculated using maximum parsimony based on the Manhattan distance of SNPs and INDELs combined (SNP/INDEL evolutionary rates are not known a priori; fig. 2 and supplementary fig. S2, Supplementary Material online).

A Lento/bipartition plot (Lento et al. 1995) was generated for this phylogeny (supplementary fig. S3 and table ST2, Supplementary Material online); for each bipartition of the tree, we compared the proportion of variants that supported each bipartition, contradicted it (requiring a homoplasy, reversion or recombination to explain the split), or were irrelevant (e.g., a variant contained entirely within one clade). The mean proportion of variants that support the phylogeny of the largest clade was 0.75. This result is consistent with a high specificity and lower sensitivity genotyping method and does not suggest any recombination has taken place in these samples. Despite the relatively short sampling times covered (only 6 years in the largest clade) bootstrap support for the DISCOVAR phylogeny is well above 50% for most within-clade relationships (mean bootstrap value 0.71); samples with low bootstrap support were separated by no more than a year.

### De Novo Variants Can Distinguish IBD Parasites

For subsequent analyses, we have worked under the assumption that the samples within clades have not undergone meiotic outcrossing with other lineages from the same clade. The Thiès region is characterized by low transmission and a previous study of 974 samples from this region, covering the same period of time, showed a low prevalence of complex (multilineage) infections (Galinsky et al. 2015). Although we cannot absolutely rule out recombination between IBD individuals, complex infections are a prerequisite for recombination. Given the low population prevalence of parasites belonging to each clade, and the attendant low probability that a single host would have been simultaneously infected by two parasite lineages of the same clade, we conclude that outcrossing is unlikely to have occurred. Variants found within a clonal expansion are therefore likely to represent de novo mutation. Of the 26,696 variants that distinguished the three clades from each other, 3,200 segregated within the clades (622, 828, and 1858 for clades 24, 26, and 29, respectively). Some loci segregated within more than one clade (9 loci in clades 24 and 26; 39 in 24 and 29; 57 in 26 and 29; 1 locus segregated in all three clades) (supplementary fig. S2, Supplementary Material online); though many of these loci showed different alleles in different clades, we are unable to distinguish between independent mutations versus shared variation. As might be expected, variants in the extended genome showed a higher degree of miscalling, with each clonal lineage having a different degree of miscalling—indicating a haplotype specific effect (supplementary table ST1, Supplementary Material online). Genes that are frequently used for the study of parasite diversity were also examined; no variants of any type were found within any of the clades for *MSP1*, *MSP2*, SERA2, *TRAP*, or *CSP* consistent with the earlier sequencing carried out on these samples (A. Bei—*personal communication*). The uniformity of these markers within an otherwise panmictic population indicated that each clade descended from a single common ancestor without recombination, the clades were therefore considered identical by descent (IBD). Within clades, pairwise distances ranged from 88 to 677 variants, with both extremes being present in clade 29. As might be expected, the oldest sample (Th086.07, collected in 2007) exhibited a greater genetic distance with respect to the other samples in clade 29, though this pattern was not repeated in the other two clades consisting of more closely contemporaneous samples (2012–2013; fig. 2).

Variants were found predominantly in intergenic sequence for both SNPs (78.5%) and INDELs (83.7%); within coding sequence in-frame INDELs and missense SNPs generate a further 15% of the variants in each class (table 1) indicating that selection would not significantly confound phylogeny reconstruction. Perhaps surprisingly, whereas we would expect the majority of sequencing errors to be observed as singletons, the proportions of nonsense variants did not increase when considering only singletons, indeed the proportion of intergenic variants significantly increased when considering only singletons. This could represent terminal-branch variants in the rapidly mutating intergenic regions or could imply that false positive variants deriving from alignment error (which would be *less* likely to be found as singletons) are more prevalent in coding regions.

**Table 1.** Mutation Consequences by Variant Class and Allele Frequency.

Variant Consequence	INDEL	SNP	Nonsingleton	Singleton
frameshift_variant	12 (1.0%)	0 (0.0%)	4 (0.2%)	8 (0.3%)
inframe_deletion	87 (7.1%)	0 (0.0%)	27 (1.6%)	60 (2.4%)
inframe_insertion	85 (6.9%)	0 (0.0%)	9 (0.5%)	76 (3.0%)
intergenic	1025 (83.7%)	2331 (78.5%)	1415 (86.0%)	1941 (76.1%)
intragenic_variant	0 (0.0%)	1 (0.0%)	0 (0.0%)	1 (0.0%)
missense_variant	12 (1.0%)	445 (15.0%)	142 (8.6%)	315 (12.4%)
non_coding_exon_variant	4 (0.3%)	10 (0.3%)	3 (0.2%)	11 (0.4%)
stop_gained	0 (0.0%)	12 (0.4%)	3 (0.2%)	9 (0.4%)
synonymous_variant	0 (0.0%)	171 (5.8%)	43 (2.6%)	128 (5.0%)

### Phylogeny Shows Distinct Substructure within a Single Clonal Expansion

An “unbalanced” phylogeny can indicate the presence of super-spreader events or long infection chains (Colijn and Gardy 2014) both of which will generate more internal nodes that are connected to a single leaf descendent. Sackin and Colless indices were used to assess the phylogenies and both measures showed some elevation above expectations for a balanced tree, however, none of the clade phylogenies were found to be significantly unbalanced (Blum and François 2005), nor was the overall phylogeny of all three clades, and we cannot conclude that any of these phylogenies represents a super-spreader event or a single infection chain.

Although all of the samples within a clade are descended from a single common ancestor over the time since this recent common ancestor, we can expect some structure to have emerged within these clades. The parsimony tree (fig. 2*b*) for the largest clade 29 showed two distinct subclades, one consisting of three samples (subclade 29.1: Th117.11, Th134.11, Th106.11) and another larger clade with six samples (subclade 29.2: Th162.12, Th230.12, Th196.12, Th132.11, Th074.13, Th106.09), with further subdivisions in the second clade. We conclude from this that the two clades represent distinct clonal expansions that have persisted separately within the population for at least 4 years, despite being transmitted within the same geographical area (supplementary fig. S4, Supplementary Material online).

### Clade 29 Is a Measurably Evolving Population

It is important to establish whether or not any of these clades represent a measurably evolving population (MEP): a population in which a set of samples can be seen to have evolved between different sampling times. In an MEP much or all of the variation will have accrued de novo between the first and last sampling years rather than due to divergence from a common ancestor before sampling began; as a result, the root-to-tip distance of the resultant phylogeny should correlate with the sampling time, because samples collected later have had more opportunity to diverge from the most recent common ancestor (MRCA) (Drummond et al. 2003).

**Fig. 3.**
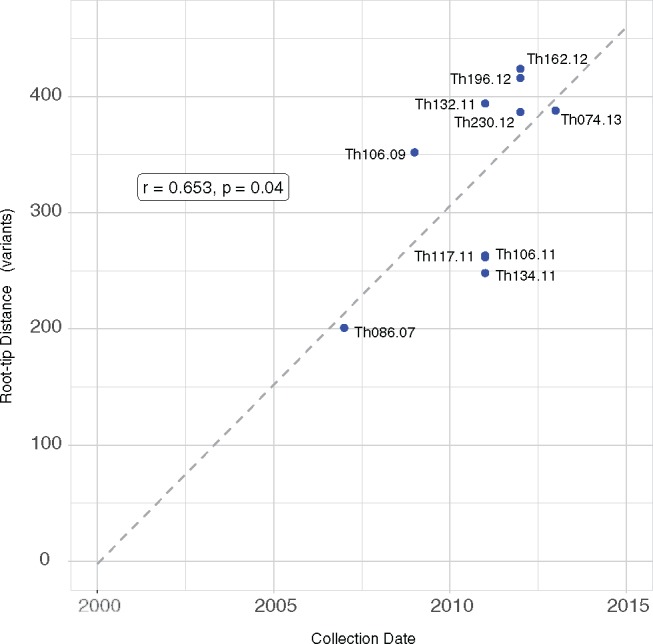
Root-to-tip distance in the phylogeny of clade 29 correlates with sampling time. The observed correlation of genetic distance and time (via Pearson’s product moment) indicates that many of our variants are de novo and that mutation occurs at a sufficiently high rate to resolve patterns of malaria transmission. Regression from sampling times indicates a common ancestor for the clade may have existed in approximately the year 2000.

The two smaller clades were not sampled over sufficient time for us to confirm that we had detected an MEP, however, clade 29 showed significant correlation between root-to-tip distance and time (*R* = 0.65, *P* = 0.04) via TempEST analysis (fig. 3) and was confirmed as being a MEP. Unlike the other two clades, clade 29 was sampled over a far greater period of time, consisting of samples from 2007 to 2013; the MEP analysis showed an MRCA in the year 2000 which, although based on a small number of samples, would imply we have sampled the clade for a little over half of the time it has been extant. Consequently this clade showed a much stronger phylogenetic signal, with TreePuzzle analysis resolving 71.4% of all quartets (supplementary fig. S5, Supplementary Material online). This clade is therefore suitable for inference of evolutionary rates and can be used to reconstruct a transmission network.

### De Novo Mutations Can Reconstruct Transmission Pathways

**Fig. 4.**
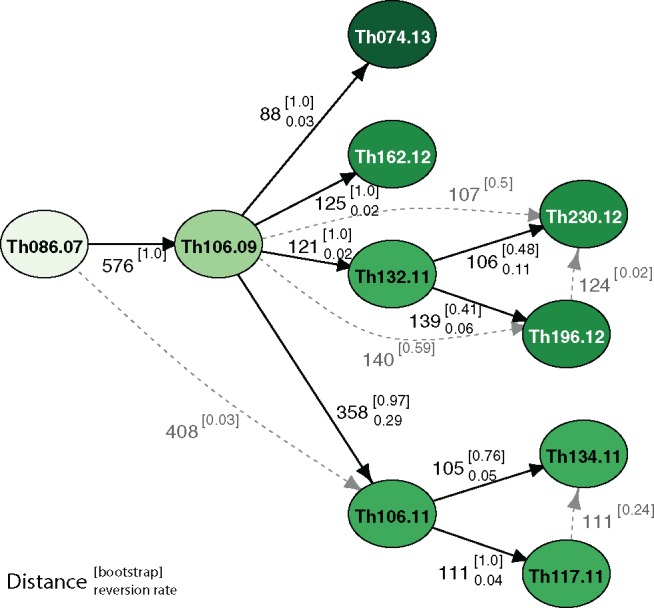
Transmission networks were estimated for clade 29 based on SNP and INDEL distances combined using a minimum distance tree approach. Edges are labeled with pairwise genetic distance, as well as bootstrap values (superscript in parentheses) and reversion rate (subscript). Bootstrap values were calculated for all edges by sampling with replacement for 100 iterations and indicate strong support for many of these distance-derived relationships. Potential alternative edges derived from the bootstrap results are shown in gray. Nodes in later years are shown in darker colors. Concordance between the parsimony tree and distance based methods is notable; in both the phylogeny and transmission network sample Th106.09 is basal to subclade 29.1 and Th106.11 to subclade 29.2. Reversion rates are significantly higher for the edge joining Th106.09 to Th106.11 supporting the independence of subclade 29.2

Transmission networks were calculated using a minimum-distance based method (Jombart et al. 2011) that relied on Manhattan distances of both variant types combined (fig. 4). Bootstrap values were high for most relationships with a majority of secondary transmission paths weakly supported. The distance-derived transmission path provided additional support for the multiclade structure that was seen in the maximum-parsimony tree results, indicating a separate expansion of subclade 29.1 and 29.2. Both of these clades were found to be related to the basal sample Th086.07.

As a secondary confirmation of the network, we mapped individual variants to each vertex in the network and calculated the reversion rate across all primary edges (i.e., the number of de novo mutations that would need to be lost to support each step); we expect that variation accrued on each lineage since divergence as well as genotyping errors will contribute to this statistic, however, these two types of “variants” would be indistinguishable. The majority of edges showed reversion rates of 11% or less, however, the edge connecting subclade 29.2 to the rest of the samples (Th106.09 through Th106.11) showed a markedly higher reversion rate (0.29). This discrepancy indicates a lack of sampling depth in the early years of the expansion and shows that we have not captured a true progenitor of subclade 29.2.

### Evolutionary Rates Indicate That De Novo Genomic Epidemiology Studies Are Viable in *P. falciparum*

**Fig. 5.**
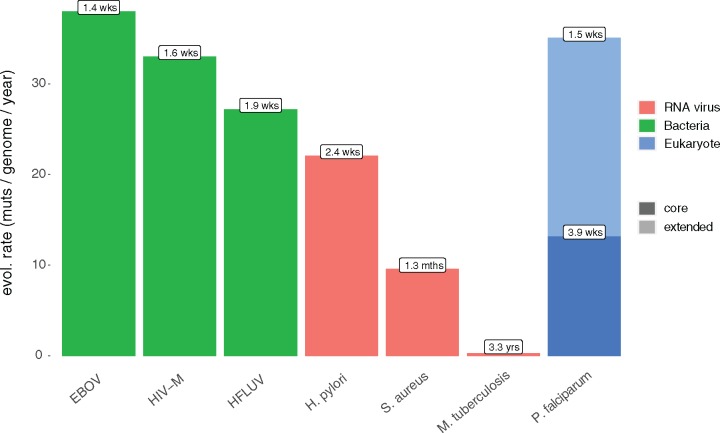
Lower mutation rates and the restricted genome size of the core genome would generate a single new mutation on average every month. The increase in accessibility offered by 250-bp reads and DISCOVAR increases both genome size and measurable evolutionary rate, resulting in a new variant every 2.4 weeks in the low complexity genome and every 1.5 weeks overall. This makes *P. falciparum* comparable to other infectious agents like influenza or HIV where transmission networks may be informed by genome sequencing. Mutation rates derived from Biek et al. (2015).

Evolutionary rates were calculated for all pairs within the transmission network for clade 29 using the method of Li et al. (1988). Highly variable evolutionary rates were seen between samples and a larger study would be necessary in order to derive a reliable evolutionary rate for *P. falciparum*. However, comparisons of the evolutionary rate within core and extended regions of the genome do show an increase in our ability to discern an MEP; the core genome acquires 0.84 (±1.8) mutations/month/genome while the extended regions gain de novo variants at three times this rate 2.08 (±1.3) muts/mth/gen (supplementary fig. S6*a*, Supplementary Material online). The combined evolutionary rate for all mutation classes is therefore 2.92 (±2.3) muts/mth/gen (fig. 5 and supplementary fig. S6*b*, Supplementary Material online). This figure is comparable to the high rate of mutation acquisition in some RNA viruses (fig. 5 and supplementary fig. S7, Supplementary Material online) and indicates that direct transmissions typically acquire at least some de novo mutations between host infections.

## Discussion

This study demonstrates for the first time the viability of using de novo mutation to characterize transmission in a eukaryotic pathogen and further shows how this technique could be used to study the transmission of malaria—a disease that, despite a worldwide elimination campaign, still kills >400,000 people per year (WHO 2016). To access rapidly evolving low-complexity sequence, we have used long sequencing reads, PCR-free library preparation and a large *k*-mer during the assembly stage, opening up a further 14% of the *P. falciparum* genome to accurate genotyping. The increased coverage in turn enables more rapidly mutating variants to be called than was previously possible, and the accompanying increased specificity prevents the de novo signal from being lost amidst the noise of sequencing or alignment error. Within a set of closely related samples from patients in Senegal (Daniels et al. 2015), we were able to call 3,200 variants that successfully resolved ancestry among these parasites and generated robust phylogenies for what appeared to be identical clones when assayed at lower genetic resolution.

Two of the clades we studied were sampled across only 2 years each and would not have the capacity to show the correlation between sampling time and root-to-tip distance that would suggest these mutations had arisen during the sampling period. On the other hand, the third clade persisted for a total of 7 years and was determined to be a measurably evolving population. This level of resolution is a first for a eukaryotic pathogen over time scales relevant to disease transmission (Biek et al. 2015) and supports the use of de novo variants to examine the phylodynamics of malaria in the future.

To demonstrate the utility of this approach, a transmission network was inferred for the largest clade using a minimum-distance approach (fig. 4; Jombart et al. 2011). For characterizing malaria transmission, de novo variation represents a significant advance over standing variation, which would be unable to identify directional relationships between infections.

Evolutionary rates as calculated across the third-clade transmission network imply one new mutation is acquired every 1.5 weeks (fig. 5), meaning that at least one new mutation should be found between direct transmissions. Although detecting a single mutation between individuals without any genotyping errors in a 23 Mb genome might be challenging with current sequencing technologies, this does indicate that sufficient de novo mutations to reconstruct transmission pathways can be expected in genomic epidemiology studies that were conducted over 2 years or more—times scales that are highly relevant to malaria transmission.

Although the transmission network was generated as a “proof-of-principle”, low rates of reversion indicate that, even if the number of intervening generations remains unresolved, many of the relationships identified are accurate and some firm conclusions can be drawn from the network about the nature of transmission within Senegal.

We can eliminate the possibility that these clonal parasites persist through a single chain of infections: an infection chain would generate a tree with each node joined only to its descendent (Colijn and Gardy 2014), yet both the tree and transmission network show strong internal structure with well supported distinct clades; in particular, the clade of three samples in 2011 (Th106.11, Th117.11, and Th134.11) is not directly related to the other clade found from 2011 to 2012 (Th132.11, Th196.12, and Th230.12). It is important to note that this conclusion would have been impossible to draw from a combination of standing variation and epidemiological data: although all six of these samples were found within the Thiès region, neither clade separates geographically and individual members from separate clades were found in close proximity (supplementary fig. S4, Supplementary Material online).

Perhaps more remarkably, despite 70% of the samples analyzed here having been taken in the intervening years, sample Th074.13 derives from Th106.09, and is entirely independent of the other two clades. This means that at the beginning of the dry season in 2011 (when transmission would be expected to tail off) at least three separate clades of this particular strain were circulating in the Thiès region and all survived to subsequent wet seasons. Maintenance of these parasite types suggests a sizable asymptomatic reservoir of infection may have been present.

Intriguingly, the smallest of those clades consists of just one sample Th074.13; this sample came from a village 16 km outside of Thiès and is separated from its closest node in the network by 6 years (supplemenatary fig. S4, Supplementary Material online), yet it is separated from this parasite by just 88 variants—the shortest genetic distance in our data set found over the longest intervening time and distance. As a result of the large population sizes that *P. falciparum* reaches during the erythrocytic life stages we do not expect de novo mutations to be commonly fixed within the host, but instead expect that differences in the consensus sequences of related infections will only be fixed by passage through population bottlenecks during mosquito transmission. The lower evolutionary rate found between these two samples could be a result of long-term asymptomatic infections during which no new variants would expect to be fixed. Further investigations of the relationship between evolutionary rate and serial interval would be of great interest and may ultimately allow us to use genetic distance to infer the duration of asymptomatic infections and their contribution to local disease incidence.

Although calculations of TMRCA are difficult from such a small sample set, the placement of the basal node in the year 2000, the diversity contained within the phylogeny, and the number of persisting clades all suggest that this clonal lineage has been long-lasting and did not emerge with sample Th086.07 in 2007.

Even with this limited data set we have successfully derived conclusions about the patterns of disease transmission in this region, but the capacity to easily derive transmission networks could be transformative for larger data sets, particularly in preelimination settings. Reiner *et al.* (2015) have demonstrated the capacity for network reconstruction to enable assessments of “malariogenic potential” (a combined measure of disease importation rates and capacity for local transmission) in Swaziland, a country nearing elimination. The network generated by Reiner et al. is derived from probabilistic modeling of transmission based upon the times and geographic locations of the infections and would enable public health authorities to target interventions such as focal mass drug administration (FMDA) where they are most needed. It is worth noting that our network inference indicates that some samples will confound expectations of spatiotemporal proximity, and validation with genetic data would be a valuable addition to such mapping attempts.

In other pathogens network reconstruction using de novo variation has already led to actionable results for infectious-disease control and these situations could apply for malaria. There are now numerous cases in which whole-genome sequencing and network reconstruction have been deployed in real time to identify the source of a hospital-acquired infection leading directly to the identification of reservoirs of persistent disease transmission (Halachev et al. 2014; Quick et al. 2015). Even on a wider geographic scale, where comprehensive sampling of an outbreak cannot be guaranteed, the approach demonstrated here can generate concrete recommendations for public health authorities. Transmission-network reconstructions of a tuberculosis outbreak in Canada were able to show that later cases were progressions from latent to active disease, rather than active transmission, with the direct result that the outbreak was declared over, a conclusion that was reached solely by comparison of the rate of de novo mutation between samples (Hatherell et al. 2016). Adapting such a capacity to malaria would have major implications for elimination efforts: malaria elimination within a country is defined as zero incidence of indigenous cases (WHO 2016) and the ability to distinguish a chain of transmission from repeated importations without recourse to a multicountry data set could be a key capacity for the WHO Global Malaria Program.

In summary, we have shown a eukaryotic parasite to be a measurably evolving population across time scales that are relevant to disease transmission, and we have calculated evolutionary rates that indicate de novo mutations could be found even between direct infections. This type of genetic signal is qualitatively different to those that have been available in previous genomic epidemiology studies in malaria and will enable finer grained characterization of disease transmission. Though this data set is relatively small, we are limited by the requirement to use only clonal outbreaks for the network inference and challenges remain before these techniques can be used in larger data sets from higher-transmission settings, where sexual recombination will add complexity to the reconstruction. We hope that future data sets revealing de novo mutations in larger collections of patient samples will motivate the development of more sophisticated methods for transmission-network reconstruction; methods that could work on recombined samples or could tolerate a lower degree of genotyping specificity. Nevertheless, there are many regions in which the techniques employed here are of immediate use. As of the end of 2015, the WHO has certified 27 countries as malaria-free, three more countries are in the process of certification (having had 3 years without indigenous transmission), and a further ten countries have had at least 1 year without indigenous transmission (WHO 2016). In any of these settings there could be outbreaks that are partially or entirely clonal. Reconstruction of a transmission network in the manner that we have achieved here would be able to detect any links that were entirely within-country, thus distinguishing imported from indigenous transmission. These data could also identify “super-spreaders” that might be targeted with focal interventions. The capacity to distinguish between indigenous and imported transmission or to pinpoint hotspots in countries nearing elimination could be key capacities for malaria elimination efforts.

## Materials and Methods

### Lab Strain Sample Preparation

DISCOVAR libraries were prepared for two Dd2 lines (Dd2-2D4 and Dd2-B2) and one 3D7 line (3D7-A-10). The Dd2-2D4 line is derived from clone MRA-156 (originally deposited by Thomas E. Wellems and available from BEI resources, NIAID, NIH https://www.beiresources.org/Catalog/BEIParasiticProtozoa/MRA-156.aspx) whereas clones Dd2-B2 and 3D7-A10 were derived from the MALDA/MDTIP consortium (Cowell et al. 2018) and are available on request. Although the Dd2 lines derive from a common subcloned patient isolate, small numbers of de novo variants are expected to have been acquired via mutation and genetic drift in the years since the laboratory lines diverged. Three biological replicates (using separate isolates of DNA) were prepared for the Dd2-B2 and 3D7-A10 samples, and one library of the Dd2-2D4. All libraries were sequenced in duplicate sequencing lanes allowing us to remove individual miscalled bases where they were not consistent across lanes.

### Senegal Sample Preparation

Samples used in this study derive from prior collections (Daniels et al. 2013, 2015) in Thiès, Senegal. All samples were collected from individuals after informed consent of either the subject or a parent/guardian. This protocol was reviewed and approved by the ethical committees of the Senegal Ministry of Health (Senegal) and the Harvard T. H. Chan School of Public Health (16330, 2008) as detailed in Daniels et al. (2015). Samples were collected passively from patients reporting to the “*Service de Lutte Anti-Parasitaire*” (SLAP) clinic for suspected uncomplicated malaria between approximately August and December each year. Patients with acute fevers or history of fever within the past 24 h of visiting the clinic and with no reported history of antimalarial use were considered; they were diagnosed with malaria based on microscopic examination of thick and thin blood smears and rapid diagnostic tests (RDT), as available. All samples were collected via venous blood draw and stored as glycerolyte stocks, and an aliquot of material collected on Whatman filter paper was extracted for nucleic acid material and genotyped via 24-SNP barcode as described in Daniels et al. (2013). From this larger data set, 18 samples were chosen representing single infections from clades 24, 26, and 29, (3, 5, and 10 samples, respectively) based on identical genotypes observed using the 24-SNP barcode. The 18 parasite samples were culture-adapted to produce parasite DNA free of host (human) contamination. Culture times were kept to a minimum (<15 cycles) to preserve the integrity of the original patient material, however, the acquisition of confounding de novo mutations within this culturing stage cannot be ruled out.

### Sequencing and Variant Discovery

DNA extracted from all samples was size selected to give a mean fragment length of 450 bp and sequenced using an Illumina HiSeq 2500 with a read length of 250 bp, ensuring an overlap of approximately 50 bp. Sequence was aligned to the Pf3D7_v3 reference using BWA-mem v. 0.7.12 (Li 2014). All alignments were deposited at the NCBI Short Read Archive (SRA) database (accession: SRP137271).

Variant discovery using DISCOVAR (release no. r52488) (Weisenfeld et al. 2014) was performed with 250 bp reads on ∼450 bp fragments. The Pf3D7 genome was subdivided into 30 kb regions and DISCOVAR was run separately for each region. In regions where it was not possible to construct a single assembly graph for variant calling, DISCOVAR was rerun with progressively smaller regions (10, 5, and 2 kb) of the genome. In all cases a 1 kb overlap was allowed to control for edge effects, though variants were only included up to the inner edge of this flanking region. All libraries were sequenced and genotyped in duplicate to remove individual miscalled bases derived from sequencing error. Resulting genotypes were filtered based on Phred score (i.e., no call above 23 or any below ten included) and hypervariable sites (those for which the genotyping software generated >50 potential alternative variants) were removed.

Variant discovery using GATK HaplotypeCaller was performed with both 250 bp reads and a more typical data set (100 bp reads with a 300 bp insert size) that was generated from the original 250 bp reads. This was followed by quality score recalibration using GATK VQSR (DePristo et al. 2011)—filtering by VQSLOD score with a 90% retention threshold. The VQSR truth-set consisted of variant loci that had been previously determined in the “*Pf*-Crosses” data set generated by Miles et al. (2016). This data set consists of a series of genetic crosses of *P. falciparum* clones (3D7 × HB3, HB3 × Dd2, and 7G8 × GB4) in which parents and all offspring were deeply sequenced, enabling variant calls to be filtered according to conformity with Mendelian inheritance expectations.

#### Variant Validation

The *P. falciparum* genome presents a challenge to many genotype callers due to extensive repeat structure and recombination within some large gene families (Bopp et al. 2013), as well as the high A/T nucleotide composition of the intergenic regions (Gardner et al. 2002). Moreover, validation of variant calls in low-complexity regions of the *P. falciparum* genome via conventional PCR and Sanger dideoxy sequencing is difficult, as PCR amplicons evaluated were generally too repetitive to yield interpretable sequence traces. As a result, a two-stage concordance-based approach to genotyping was taken: 1) the “accessible” genome was determined separately for each genotype caller; 2) genotypes in the regions accessible to both callers were compared with a known sample and assessed for specificity.

##### Accessible Genome Determination

Miles et al. used the *Pf-Crosses* data set in order to characterize the “accessible genome”—the region in which genotyping calls could be reliably made (Miles et al. 2016). The authors found 90% of the genome to be within an acceptable error rate, and only hypervariable regions, centromeres and telomeres were excluded. DISCOVAR generates few variables upon which variant recalibration could be performed; therefore, we took a stricter approach. Using in silico sequence generated from a second genome assembly, we determined a region to be accessible only if the variant caller can successfully reconstruct the region around all variants within it. Three variant calling approaches were assessed: DISCOVAR using 250 bp reads, HaplotypeCaller (DePristo et al. 2011) with 250 bp reads (HC250) and HaplotypeCaller with 100 bp reads (HC100).

An artificial (in silico) Dd2-2D4 read set (250 bp fragment size, 450 bp insert size) was generated from a PacBio assembly of the Dd2-2D4 line of *P. falciparum* (ftp://ftp.sanger.ac.uk/pub/project/pathogens/Plasmodium/falciparum/DD2/) using wgsim v0.3.2 (Li et al. 2009) using default parameters and generating 100X coverage. This read set was aligned to the standard Pf3D7_v3 reference assembly and genotypes were called using DISCOVAR and HaplotypeCaller as detailed above. For each resulting variant, the putative Dd2-2D4 sequence in a 200 bp region around the variant was reconstructed and realigned to the Dd2-2D4 PacBio reference via Smith–Waterman alignment (BWA-SW [Li and Durbin 2010]). Any differences between this locally reconstructed Dd2-2D4 sequence and the Dd2-2D4 PacBio assembly (i.e., the Levenshtein distance between the two strings) would therefore represent errors derived either from misalignment of the simulated reads or from the genotyping algorithm itself. This Smith–Waterman approach was used to compare calls made using DISCOVAR using 250 bp reads, HaplotypeCaller using the same 250 bp reads, and via HaplotypeCaller using 100 bp reads. For each variant calling method, the “accessible genome” was defined as those regions where alignment depth was within one standard deviation of the mean and where the Levenshtein distance (LD) was zero (i.e., where reconstruction of Dd2-2D4 had been error free). Low complexity regions of the genome were assessed by running Dustmasker (version 1.0 in package BLAST+ 2.2.30 using default parameters).

#### Accessible Marker Validation

An experimentally validated set of genotypes was then used for confirmation of variants within those regions of the genome that were accessible to both HaplotypeCaller and DISCOVAR. This biological validation was performed using three laboratory lines of *P. falciparum*, each sourced from different laboratories and available on request. The called variants were then compared with the set of previously validated “*Pf-Crosses*” variants using VCFEval (Cleary et al. 2015): receiver-operator curves (ROC curves—i.e., plots of true positive and false positive rates at equal quality scores) and specificity/sensitivity values were calculated in the regions that had been previously determined to be accessible by both genotype callers and in the regions determined to be accessible only to DISCOVAR. Pf-Crosses data is available from: https://www.malariagen.net/data/pf-crosses-1.0

### Phylogeny and Transmission Networks

SNP and INDEL distance matrices were calculated based on the number of mutations between each sample pair, without making adjustments based on putative mutation rate or STR length (all INDELs were treated as a single mutation regardless of length or whether they were found within a known STR). Parsimony trees were generated using the R package *Phangorn* (Schliep 2011) and neighbor-joining trees were generated using the R package “ape” (Paradis et al. 2004). Phylogenies were calculated by both methods for the pairwise SNP distance, INDEL distance and the Manhattan distance of both SNPs and INDELs. Bootstrapping was performed by “sampling with replacement” for 100 replicates across each phylogeny. Bootstrap support is reported as the proportion of trees supporting each node.

To assess the phylogenetic signal within the clades, the Tree-Puzzle program (Schmidt et al. 2002) was used to analyze all potential sequence quartets within the set. For any set of four individuals, a maximum of three phylogenies are possible, and the TreePuzzle method randomly selects individuals from the data set and assesses by maximum likelihood if it accords with any of the three phylogenies (see Strimmer et al. for details [Strimmer and von Haeseler 1996]). A quartet with clean phylogenetic signal will show high likelihood to one of the tree topologies, a network with significant introgression will show likelihood to more than one, and an unresolved star-like phylogeny will show likelihood to none.

Temporal data were assessed by examining the correlation between sampling time and the root-to-tip distance of a “non-clock” phylogenetic tree (in this case one derived by neighbor-joining) using TempEST v1.5 (Rambaut et al. 2016). Correlation between genetic distance and time was assessed via Pearson’s product moment. Transmission networks were reconstructed using SeqTrack within adegenet (Jombart et al. 2011) based on the Manhattan distance of all variants: an approach that has been shown to outperform parsimony based methods where sampling density is low (Worby et al. 2017). Bootstrapping of the transmission network was performed by sampling all variants with replacement for 100 iterations; bootstrap values are reported as the proportion of bootstrapped networks supporting each join.

### Evolutionary Rate Calculation

For samples sharing very recent common ancestry, pairwise variant call differences are expected to be dominated by sequencing error, leading to overestimation of the rate at which de novo mutations accumulate. Where a transmission network could be resolved for a clade, evolutionary rates were therefore calculated for each IBD pair as the difference between the distance of each sample to an outgroup (chosen from one of the other two clades) to account for ancestral diversity (Li et al. 1988). This was performed separately for the regions of the genome determined to be callable with DISCOVAR, HC250, and HC100 and the DISCOVAR-only genome yielding an estimate of the contribution to the evolutionary rate of “core” and “extended” regions of the genome. The mean evolutionary rate is reported for each pair of samples using all potential outgroups.

## Supplementary Material

Supplementary data are available at *Molecular Biology and Evolution* online.

## Supplementary Material

Supplementary Data
