# Supplementary material for: De Novo Mutations Resolve Disease Transmission Pathways in Clonal Malaria
Source: Mol Biol Evol. 2018 May 1;35(7):1678–89. doi: 10.1093/molbev/msy059 (PMC5995194; doi:10.1093/molbev/msy059)
Supplement: Supplementary Data [file msy059_supp.docx]

Supplemental figures:

Fig S1: ROC plots, core/extended genome


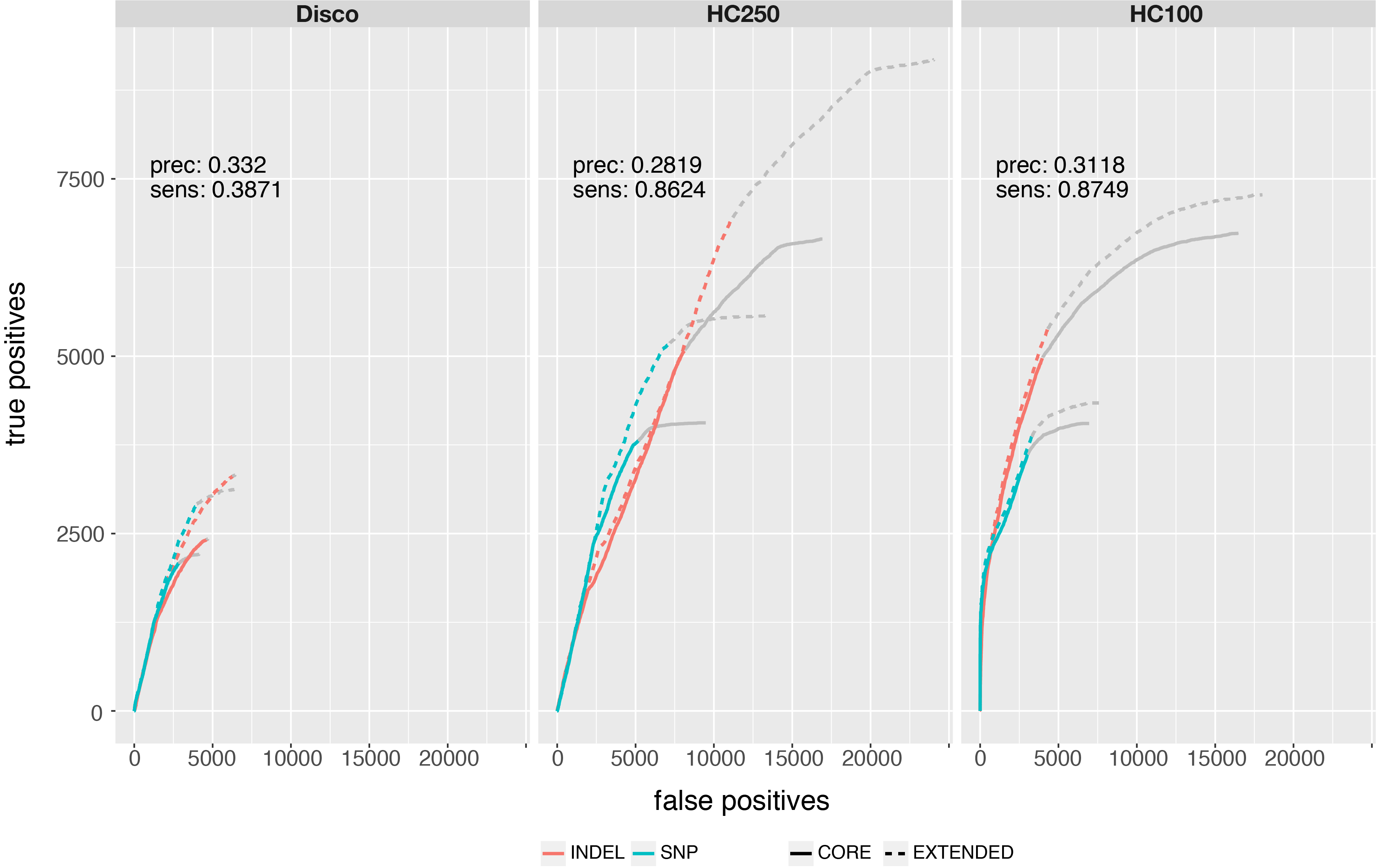


**Fig S1:** ROC curves for DISCOVAR-called variants with 250 bp reads (a), GATK HaplotypeCaller variants with 250 bp reads (b) and HaplotypeCaller with 100 bp reads (c). Variants were called in the ‘core’ genome that is accessible to all callers, as well as the ‘extended’ genome – the entire genome that is accessible to that caller. Variants below the VQSLOD cutoff threshold are colored in gray. DISCOVAR demonstrated reduced sensitivity but higher specificity compared to GATK HaplotypeCaller.

Fig S2: *De novo* SNP / INDEL distance matrices


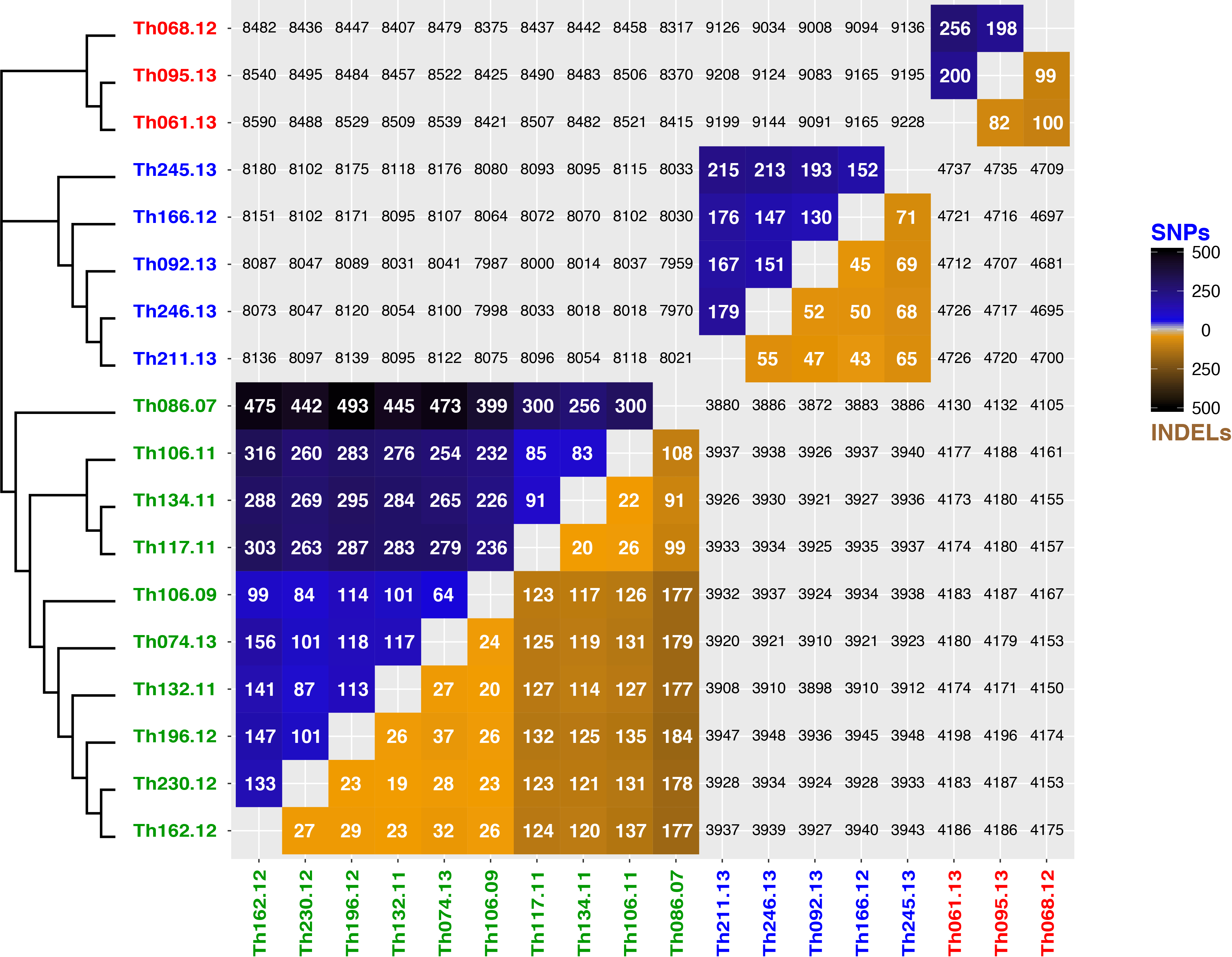


**Fig S2:** More than 3000 de novo variants were called via DISCOVAR that varied among the individuals in the three IBD clades. Clades 24, 26 and 29 were separated by 622, 828 and 1858 variants respectively, with the closest individuals (Th106.09 / Th074.13) separated by 88 de novo variants. The parsimony-derived cladogram is shown alongside.

Fig S3: Lento Plots / Phylogeny variant support


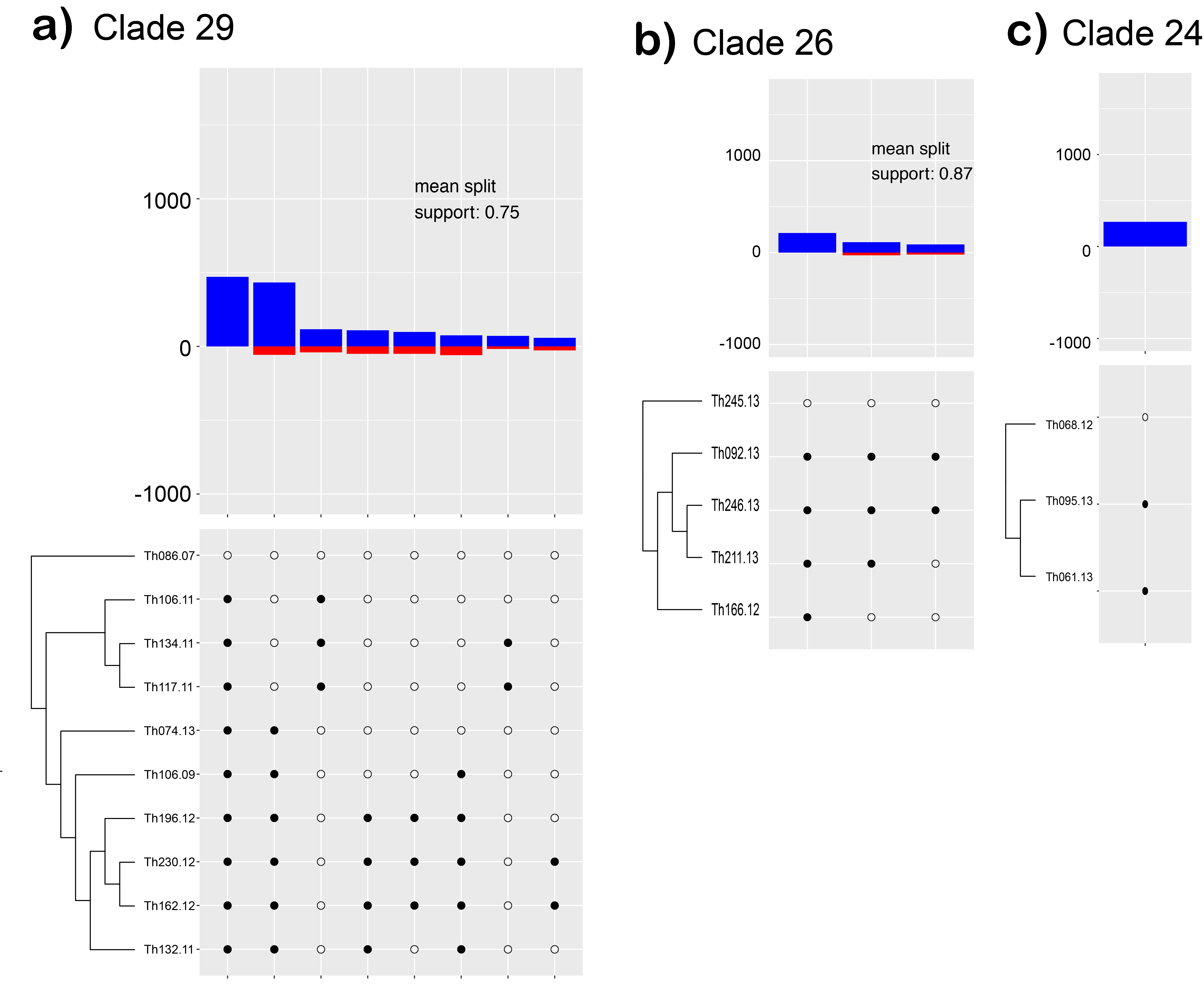


**Fig S3:** Lento plot showing the number of variants perfectly supporting or conflicting with each split within the DISCOVAR derived trees for clades a) 29 b) 26 and c) 23. Supporting variants are shown in blue, conflicting (on the negative y axis) in red. Variants that neither support nor conflict (e.g, singletons or those that are entirely within one of the splits) are not shown. Strong support is seen for all of the nodes in the maximum-parsimony tree. Once singletons and otherwise uninformative markers are removed, between 59-100% of all variants support each node (see Table ST1).

Figure S4: geographical transmission network, Clade 29


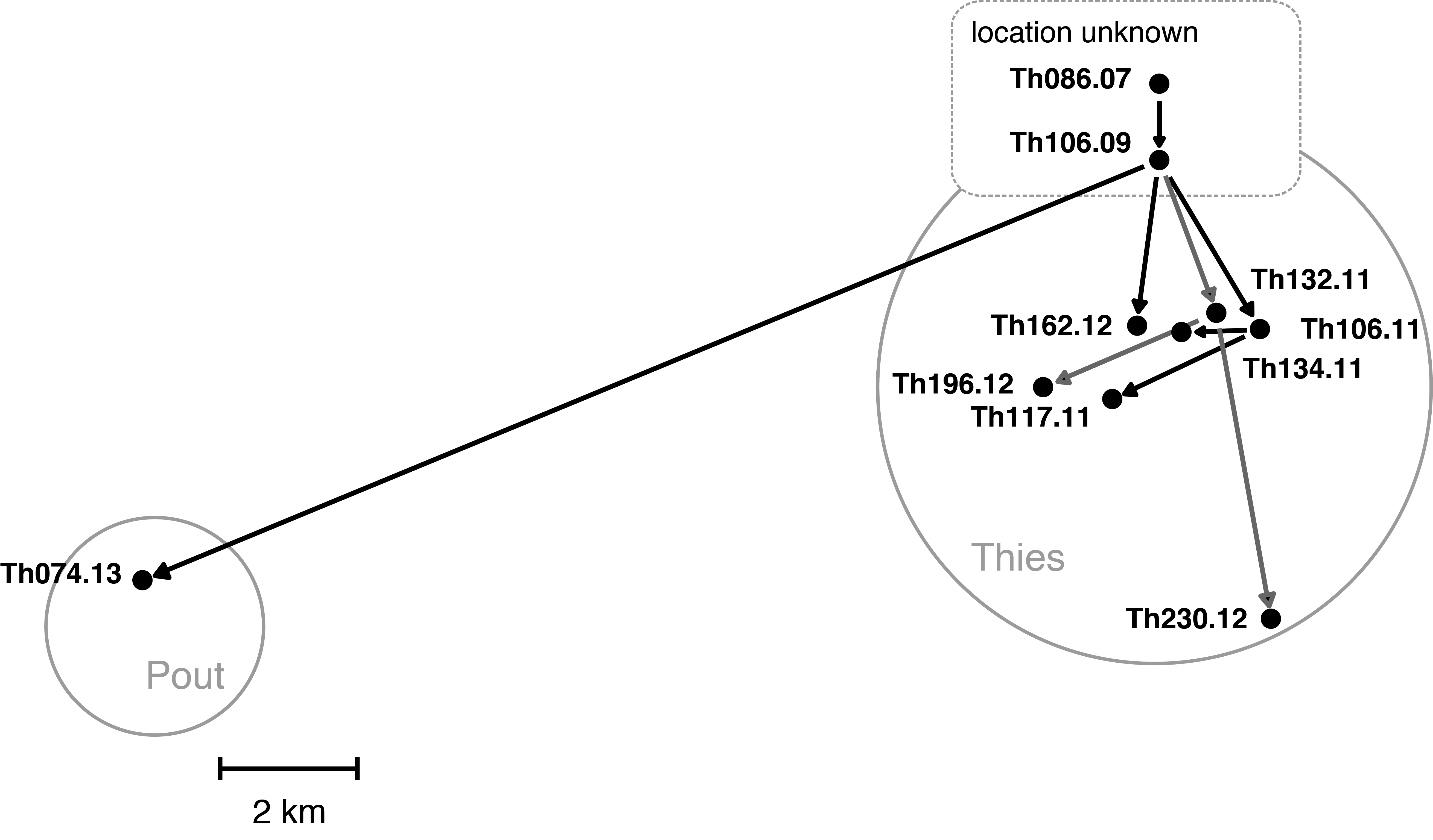


**Fig S4:** The transmission network can be plotted using self-reported home locations for each patient. Locations are reported as the center of each neighborhood, rounded to the nearest 100^th^ of a degree. The underlying map is not shown to preserve patient confidentiality, though the outlines of Thies and Pout are given. Two samples (Th086.07 and Th106.09) did not report a home address. Two entirely separate clades of 3 infections are seen within Thiès between the years of 2011-2012; in many if not most cases, an infection does not transmit to the immediately proximal patient, implying longer vector or human travel distances are ablating any link between genetic and geographic distance.

Fig S5: Tree-Puzzle analyses

c)


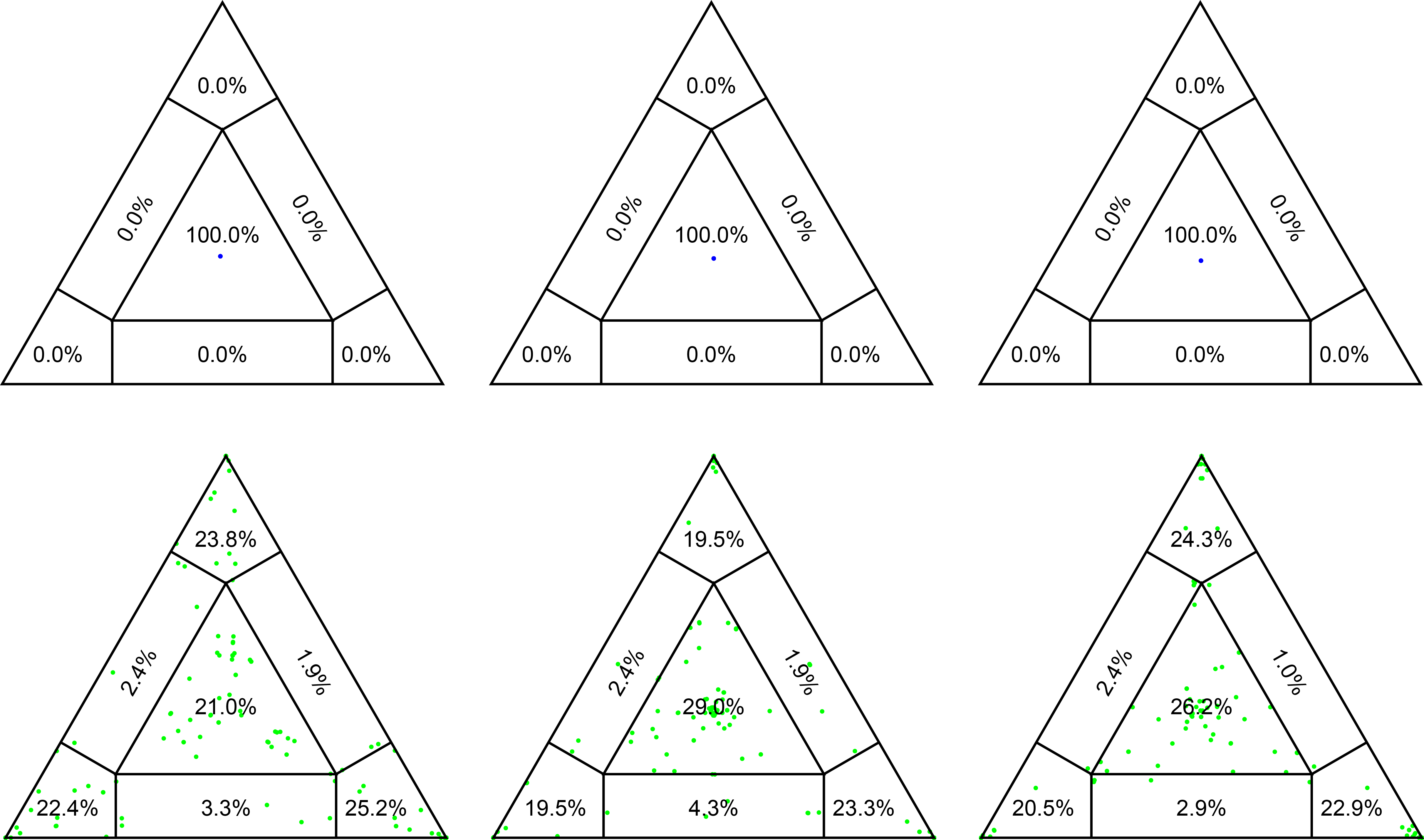


f)

e)

d)

b)

a)

**Fig S5:** Where sufficient samples were available we ran tree-puzzle analyses for SNPs only, INDELs only, and all variants. Tree-Puzzle plots are given for clade 26: SNPs (a) INDELs (b) and all variants (c), and for clade 29 on the same variant classes (d-f). We were unable to perform a Tree-Puzzle analysis for clade 24 since it had fewer than the minimum four samples and clade 26 did not show strong support for the phylogeny. However the results for clade 29 indicated a strong phylogenetic signal within the dataset, with 71.4%, 62.3%, and 67.7% of all quartets fully resolved in ALL, SNPs and INDELs respectively.

Fig S6: Evolutionary Rates


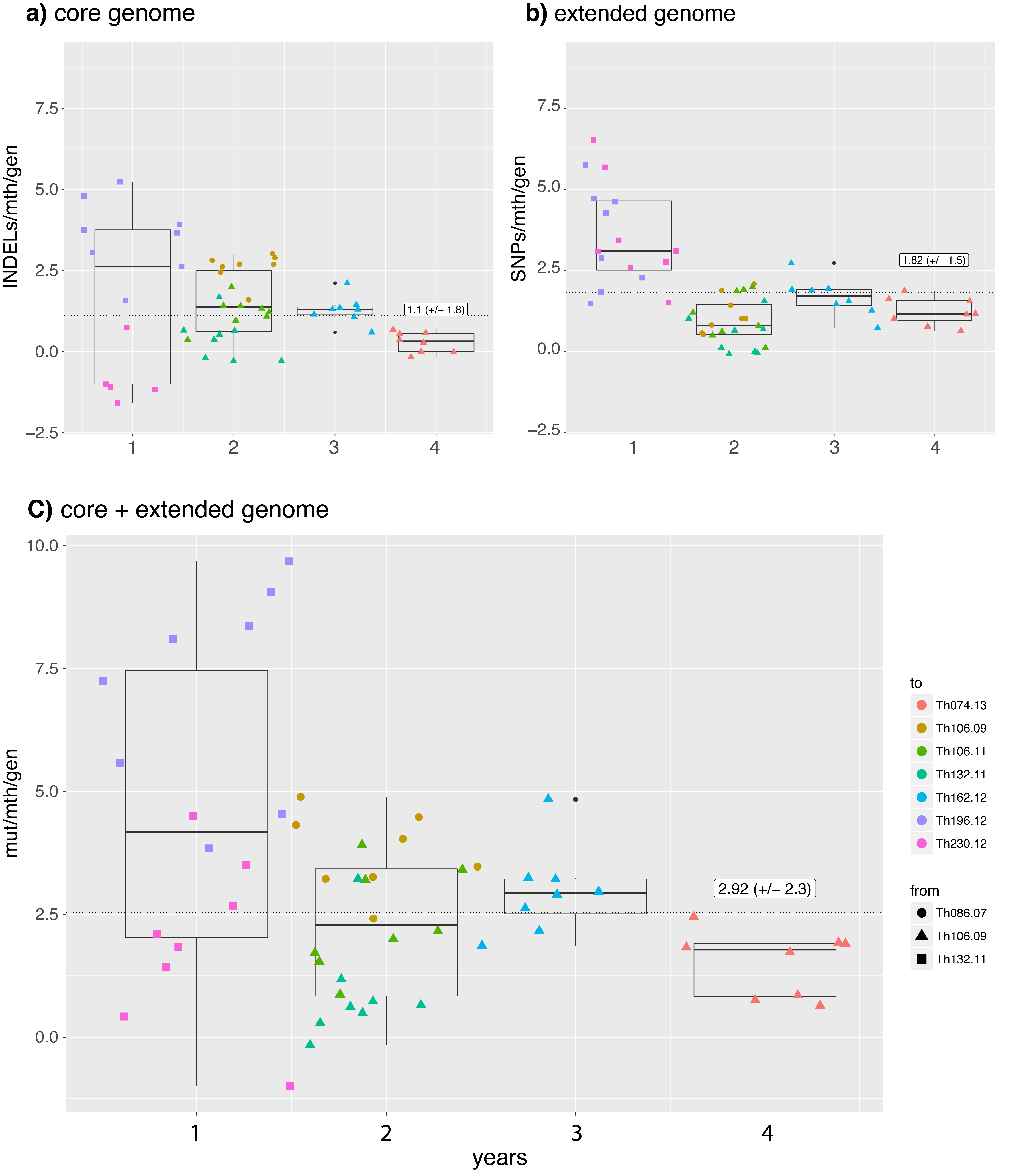


**Fig S6:** Evolutionary rates were calculated by comparing, for each pair of IBD samples, the difference between pairwise distances of each to an outgroup. Outgroups were chosen from the members of the two other clades and all rates are shown on the plot above. Separate evolutionary rates were calculated for the core genome (a), the extended genome (b) and both core and extended regions (c) Despite high variances in many samples deriving from divergence from individual outgroups, and some negative rate values (where the parent had diverged further from the outgroup than the child) samples separated by more than 3 years showed little variation in evolutionary rate. The extended genome is responsible for the majority of this rapid evolutionary rate, showing around 3x the evolutionary rate of the ‘core’ genome.

Fig S7 Mutation rates vs. genome size


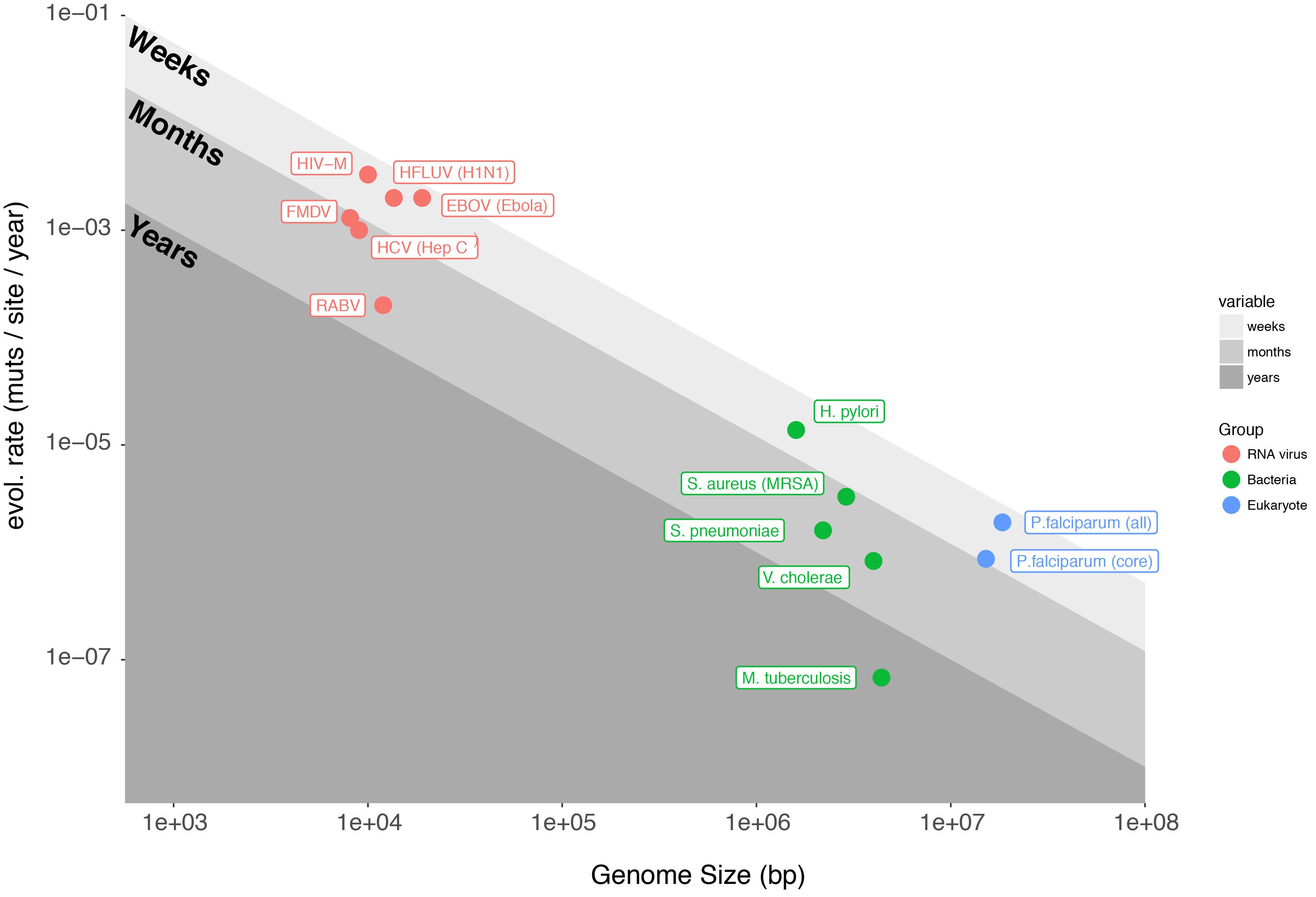


**Fig S7:** viability of genomic epidemiology via de novo variant detection is determined by a combination of genome size and mutation rate. Parasites with an extremely rapid mutation rate can be viable despite the small genome size offering few opportunities to acquire new mutation. The increase in accessibility we have achieved increases both genome size and detectable evolutionary rate, and places P.falciparum in the same class as H pylori or HFLUV for genomic epidemiology studies. (Adapted from Biek et al. 2015)

Table ST1: Coverage and missing-genotype rates for all clinical samples

|  |  |  | Core (N = 13589) | | Extended (N = 35940 bp) | |
| --- | --- | --- | --- | --- | --- | --- |
| Sample | Clade | >5x coverage (bp) | No. missing (bp) | Freq. missing (%) | No. missing (bp) | Freq. missing (%) |
| Th086.07 | 29 | 21,633,850 | 278 | 0.02 | 2524 | 0.07 |
| Th106.09 | 29 | 22,728,816 | 343 | 0.03 | 1997 | 0.06 |
| Th106.11 | 29 | 22,688,773 | 262 | 0.02 | 1908 | 0.05 |
| Th117.11 | 29 | 22,655,106 | 305 | 0.02 | 1699 | 0.05 |
| Th132.11 | 29 | 22,681,144 | 321 | 0.02 | 1920 | 0.05 |
| Th134.11 | 29 | 22,612,620 | 319 | 0.02 | 1917 | 0.05 |
| Th162.12 | 29 | 22,413,921 | 327 | 0.02 | 1885 | 0.05 |
| Th196.12 | 29 | 22,670,792 | 278 | 0.02 | 1788 | 0.05 |
| Th230.12 | 29 | 22,701,771 | 336 | 0.02 | 1782 | 0.05 |
| Th074.13 | 29 | 22,717,476 | 293 | 0.02 | 1663 | 0.05 |
| Th166.12 | 26 | 22,858,642 | 484 | 0.04 | 4871 | 0.12 |
| Th092.13 | 26 | 22,900,997 | 541 | 0.04 | 4874 | 0.14 |
| Th211.13 | 26 | 22,873,647 | 408 | 0.03 | 4607 | 0.13 |
| Th245.13 | 26 | 22,943,682 | 385 | 0.03 | 4576 | 0.13 |
| Th246.13 | 26 | 22,894,596 | 430 | 0.03 | 4819 | 0.13 |
| Th068.12 | 24 | 22,799,541 | 516 | 0.04 | 4759 | 0.13 |
| Th061.13 | 24 | 22,813,547 | 471 | 0.03 | 4625 | 0.13 |
| Th095.13 | 24 | 22,768,157 | 426 | 0.03 | 4551 | 0.13 |

Table ST2: Proportional support of all variants for each split within the Maximum Parsimony phylogeny

|  |  | DISCOVAR |  |  | HC250 |  |  | HC100 |  |  |
| --- | --- | --- | --- | --- | --- | --- | --- | --- | --- | --- |
| clade | split | irrelevant | pro | anti | irrelevant | pro | anti | irrelevant | pro | anti |
| 29 | 1 | 0.69 | 0.31 | 0.00 | 0.54 | 0.23 | 0.23 | 0.53 | 0.47 | 0.00 |
| 29 | 2 | 0.70 | 0.26 | 0.03 | 0.75 | 0.01 | 0.24 | 0.65 | 0.13 | 0.22 |
| 29 | 3 | 0.92 | 0.05 | 0.04 | 0.76 | 0.01 | 0.23 | 0.77 | 0.01 | 0.22 |
| 29 | 4 | 0.90 | 0.07 | 0.03 | 0.81 | 0.01 | 0.18 | 0.8 | 0.01 | 0.2 |
| 29 | 5 | 0.91 | 0.06 | 0.03 | 0.87 | 0.03 | 0.10 | 0.88 | 0.01 | 0.11 |
| 29 | 6 | 0.95 | 0.04 | 0.02 | 0.54 | 0.23 | 0.23 | 0.89 | 0.00 | 0.11 |
| 29 | 7 | 0.90 | 0.07 | 0.02 | 0.79 | 0.04 | 0.17 | 0.82 | 0.03 | 0.15 |
| 29 | 8 | 0.94 | 0.05 | 0.01 | 0.86 | 0.03 | 0.12 | 0.89 | 0.01 | 0.11 |
| 26 | 1 | 0.68 | 0.32 | 0.00 | 0.83 | 0.17 | 0.00 | 0.81 | 0.19 | 0.00 |
| 26 | 2 | 0.80 | 0.16 | 0.04 | 0.69 | 0.15 | 0.15 | 0.67 | 0.11 | 0.22 |
| 26 | 3 | 0.85 | 0.12 | 0.03 | 0.70 | 0.10 | 0.20 | 0.70 | 0.16 | 0.14 |
| 24 | 1 | 0.50 | 0.50 | 0.00 | 0.49 | 0.51 | 0.00 | 0.59 | 0.41 | 0.00 |
